# Health status in routine clinical practice: validity of the clinical COPD questionnaire at the individual patient level

**DOI:** 10.1186/1477-7525-8-135

**Published:** 2010-11-16

**Authors:** Janwillem WH Kocks, Huib AM Kerstjens, Sandra L Snijders, Barbara de Vos, Jacqueline J Biermann, Peter van Hengel, Jaap H Strijbos, Henk EP Bosveld, Thys van der Molen

**Affiliations:** 1 Department of General Practice, University Medical Center Groningen, University of Groningen, A. Deusinglaan 1, 9713 AV Groningen, the Netherlands; 2 Groningen Research Institute for Asthma and COPD (GRIAC), University Medical Center Groningen, Hanzeplein 1, 9700 AW Groningen, the Netherlands; 3 Department of Pulmonary Diseases and Tuberculosis, University Medical Center Groningen, Hanzeplein 1, 9700 AW Groningen, the Netherlands; 4 Department of Pulmonary Diseases and Tuberculosis, Wilhelmina Hospital, Postbus 3000, 9400 RA Assen, the Netherlands; 5 Department of Pulmonary Diseases and Tuberculosis, Nij Smellinghe Hospital, Postbus 20200, 9200 DA Drachten, the Netherlands

## Abstract

**Background:**

There is a growing interest to use health status or disease control questionnaires in routine clinical practice. However, the validity of most questionnaires is established using techniques developed for group level validation. This study examines a new method, using patient interviews, to validate a short health status questionnaire, the Clinical COPD Questionnaire (CCQ), at the individual patient level.

**Methods:**

Patients with COPD who visited an outpatient clinic completed the CCQ before the consultation, and the specialist physician completed it after the consultation. After the consultation all patients had a semi-structured in-depth interview. The patients' CCQ scores were compared with those of the treating clinician, and with mean scores from 5 clinicians from a pool of 20 who scored the CCQ after reading the transcript of the in-depth interviews only. Agreement was assessed using Lin's concordance correlation coefficient (CCC), and Blant and Altman plots. Interviews with patients with low agreement were reviewed for possible explanations.

**Results:**

A total of 44 COPD patients (32 male, mean age 66 years, FEV_1 _45% of predicted) participated. Agreement between the patients' CCQ scores and those of the treating clinicians (CCC = 0.87) and the mean score of the reviewing clinicians (CCC = 0.86) was very high. No systematic error was detected. No explanation for individuals with low agreement was found.

**Conclusion:**

The validity of the CCQ on the individual patient level, as assessed by these methods, is good. Individual health status assessment with the CCQ is therefore sufficiently accurate to be used in routine clinical practice.

## Background

Health status measurement by questionnaires can be used in routine clinical practice to enhance communication, monitor disease progression and response to treatment, screen for undetected disability, improve patient satisfaction, and assess disease severity [1,2]. Questionnaires available for use in routine clinical practice must be short, and easy to administer, score and interpret; in addition, guidelines for their interpretation should be available [3,4]. Questionnaires should also be reliable and validated for the patient who completes the questionnaire.

Methods to develop and validate health status or quality of life questionnaires are well established. These validation processes focus on their use in clinical trials in groups of patients. However, despite their increasing use in everyday practice, we found only one proposed guideline for the validation of questionnaires in the individual patient. In 1995 McHorney and Tarlov suggested a number of measurement standards for individual patient application of questionnaires, such as high internal consistency reliability (above 0.9) and a small standard error of measurement, besides usual qualities such as construct validity and sensitivity to clinical change [3]. Although these proposed standards are mainly based on current knowledge and 'common sense', practically no questionnaires have been validated for individual health status assessment according to these standards [3].

Reliability levels of 0.90-0.95 are difficult to meet for many existing questionnaires. Secondly, since reliability is related to questionnaire length and measuring a unidimensional construct, newly developed questionnaires aiming to achieve these levels of reliability should be long and unidimensional (i.e. they measure only one aspect of the disease). However, clinicians might be more interested in being informed about several aspects of the disease (e.g. emotions, functional state and symptoms) and may prefer short questionnaires. Therefore, it may be interesting to ignore these suggested guidelines and assess whether a questionnaire is valid according to the dynamics of routine clinical practice.

To address this question we published a proposal for a new methodology [5]. In this methodology the patient's health status in daily life (as measured by an in-depth interview) is used as the gold standard, and the outcome of the in-depth interview is compared with the patient's score on the questionnaire completed in the clinic before the in-depth interview took place. We applied this new methodology in patients with chronic obstructive pulmonary disease (COPD), a condition that has a large impact on health status [6], even in mild disease [7].

One of the health status questionnaires used in COPD in clinical trials and clinical practice is the Clinical COPD Questionnaire (CCQ) [8]. This is a short 10-item questionnaire with answers based on a 7-point Likert scale. The final score is calculated by simply summing the item scores and dividing them by the number of items. The CCQ has three domains: symptoms (4 items), mental health (2 items) and functional status (4 items). The CCQ has shown to be reliable health status measure, is responsive to treatment and is stable over time if no changes occur[8,9].

This article describes the validation, at the individual patient level, of the CCQ.

## Methods

### Patients

Patients with physician diagnosed COPD, and confirmed by lung function measurement, visiting an outpatient clinic were invited to participate in the study. Patients were excluded if they had suffered a myocardial infarction within 3 months prior to enrolment. All patients gave written informed consent.

### Measurements

Lung function was taken from the patient's charts, including height and weight. Exercise capacity was assessed by the 6-min walking distance test performed according to the ATS criteria [10]. Pulse oxygenation and BORG scores for dyspnoea [11] were measured before and after the walking test. Health status was measured using the CCQ. Dyspnoea during exercise was measured with the MRC dyspnoea score. The BODE score (a multidimensional index) was calculated[12].

### CCQ

The CCQ is a 10-item health status questionnaire measuring symptoms, functional status and mental status in patients with COPD. The questionnaire is self-administered, and can be completed in 2 min. The CCQ has a high internal consistency reliability (0.91 [8]) and a small standard error of measurement (0.21 [13]) The minimal clinically important difference (MCID) was calculated using three different methods and is set at 0.4 points [13].

### Study design

Patients completed the CCQ prior to the routine consultation with their pulmonary clinician. Directly after the consultation, the pulmonary clinician (without knowledge of the patient's scores) completed the CCQ as he thought the patient should have completed the CCQ. After the consultation, patients performed the 6-min walking distance test.

One of the investigators (SLS or BdV), who did not know the patient, held a semi-structured in-depth individual interview with the patients on the day of the consultation. Patients were asked to comment on every separate concept of the questionnaire. They were asked what thoughts they had during completion of the individual questions, and were asked to give examples of their symptoms and disabilities in daily life.

### Group of reviewing clinicians

All interviews were recorded and fully transcribed. All references to scores on individual items of the questionnaire (in numbers or words) were covered by black bars to blind these results for the reviewing clinicians.

Twenty sets were created containing: i) patient characteristics: gender, age, marital status, forced expiratory volume in one second (FEV_1_) %predicted, body mass index, 6-min walking distance, oxygenation at start of the 6-min walking distance, and the number of pack years; ii) the transcribed and blinded interview; and iii) a blank CCQ.

Each set of interviews contained 11 randomly assigned interviews. The order in which interviews were in the packaged set was randomised to prevent fatigue of the reviewers and learning effects in the interviews performed later in sequence.

These sets were sent to 20 pulmonary physicians and general practitioners who have a special interest in pulmonary diseases. The clinicians were instructed to complete the CCQ of a patient the way they thought the patient should have rated the CCQ, based on the patient characteristics and interview.

This method resulted in each patient/interview being reviewed and scored by five separate clinicians.

### Data processing

The agreement between patient CCQ scores and the treating physician and reviewing clinicians scores was presented in Blant and Altman plots. The Shapiro-Wilk normality test was used to assess normality.

The pairwise agreement (concordance) between patient's score, treating clinician's score and the mean of the scores of the five reviewing clinicians, was studied by two coefficients: the intraclass correlation coefficient (ICC) and Lin's concordance correlation coefficient (CCC). Both range from 0 = no agreement, to +1 = perfect agreement.

Lin et al. have proposed a unified approach for assessing agreement for continuous and categorical data [14]. For the pairwise agreement used in our study, the unified estimate reduces to the original CCC proposed by Lin [15]. The CCC contains a measurement of precision and of accuracy for a better understanding of the sources of disagreement.

The equation from Lin (1989) is

$$\rho_{c}=\frac{2s_{xy}}{s_{x}^{2}+s_{y}^{2}+{\left(\bar{x}-\bar{y}\right)}^{2}}$$

where $\bar{x}$ and $\bar{y}$ are the mean values of the measures at 2 times, by 2 raters, or by 2 methods. Lin further proposes two absolute indices, the Total Deviation Index (TDI) and the Coverage Probability (CP), which are independent of the total data range. The MCID is used for the Coverage Probability.

The cut-off points described for rating agreement based on the ICC are ≤ 0.4 as poor to fair, 0.41-0.6 as moderate, 0.61-0.8 as good, and 0.81-1.0 as excellent. Because the CCC and ICC measure the same construct, the cut-off points can be assumed to be similar.

A significance level of 0.05 was considered statistically significant.

All analyses (excluding the CCC) were performed using SPSS for windows version 14. The CCC was calculated using SAS version 9.1 for Windows and the macro available at http://tigger.uic.edu/~hedayat/

## Results

A total of 44 patients participated in the study, in equal numbers at the two locations. Most patients had severe COPD. Table 1 presents the characteristics of the study participants.

**Table 1:** Characteristics of the study population.

		Number	(%)	Mean	(± SD)	IQR (25th-75th)
**Gender**	Male	32	(72.7)			

	Female	12	(27.3)			

**Age (years)**				66.1	(7.4)	

**Marital status**	Married	33	(75.0)			

	Not Married	7	(15.9)			

	Divorced	1	(2.4)			

	Widow	3	(6.8)			

**Educational level**	No primary school	3	(6.8)			

	Primary school	10	(22.7)			

	High school	24	(54.5)			

	College/University	5	(11.4)			

	Missing	2	(4.5)			

**Pack years**				29.3	*	**(15.7-46.8)**

**FEV1 % predicted**				44.8	(13.8)	

**Tiffenau**				41.2	11.1	

**GOLD stage**	I	0	(0.0)			

	II	13	(29.5)			

	III	26	(59.1)			

	IV	5	(11.4)			

**BODE score**				2.9	(1.8)	

**CCQ Total score**				2.2	(0.9)	

**Current exacerbation**		**5**	**(11.4)**			

IQR: Inter Quartile Range, FEV1: Forced Expiratory Flow in one second

**Figure 1:**
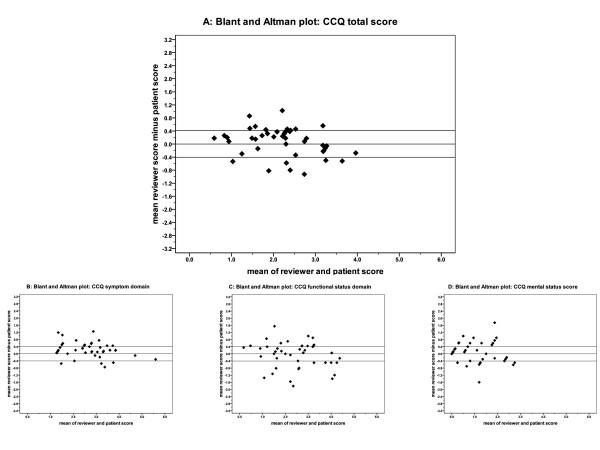
**Blant and Altman plots showing the relationship between the scores of the patients and the reviewers**. A: Clinical COPD Questionnaire (CCQ) total score; B: CCQ symptoms domain score; C: CCQ functional status domain score; D: CCQ mental state domain score.

The relation between the patient's scores and the reviewer's scores is shown in the Blant and Altman plots (Figure 1). No systematic errors can be seen as there is no trend visible. The Blant and Altman plots of the separate domains show more deviation from the origin than the total score, where the functional status and mental state have the largest deviation.

The Shapiro-Wilk normality test revealed that the total scores of the patients, the treating clinicians and the reviewing clinicians are normally distributed, guaranteeing correct confidence intervals. Table 2 shows that the agreement between patients' CCQ score and the scores of the treating clinicians (CCC = 0.87) and the mean score of five reviewing clinicians (CCC = 0.86) was excellent. The agreement between the treating clinicians and reviewing clinicians was good (CCC = 0.74). In all three cases the accuracy was considerably higher than the precision. The CCQ scores of the patients were within the limits of the MCID of the scores of the treating clinician in 62% and the mean score of the reviewing clinicians in 63%. The proportion of cases within the MCID of 0.4 (CP_0.4_) between treating clinician and reviewing clinicians was lower (0.50).

**Table 2:** Agreement between patient, treating clinician and reviewing clinicians.

Agreement between:	Statistics	CCC	Precision	Accuracy	TDI 0.9	CP 0.4
**Patient & Treating clinician**	Estimate	0.87	0.88	0.98	0.7	0.62
	
	95% Conf. Limit	0.79	0.81	0.94	0.9	0.53

**Patient & Mean reviewing clinicians**	Estimate	0.86	0.87	0.99	0.7	0.63
	
	95% Conf. Limit	0.79	0.80	0.94	0.9	0.54

**Treating clinician & Mean reviewing clinicians**	Estimate	0.74	0.76	0.97	1.0	0.50
	
	95% Conf. Limit	0.61	0.63	0.89	1.1	0.42

95% Conf. Limit: 95% confidence limit. CCC: concordance Correlation Coefficient, TDI_0.9: _A total deviation index of 0.9 (TDI 0.9) represents the distance (percentile) that captures 90% of the differences in scores between the treating clinician and the patient. A TDI 0.9 of 0.7 means that in 90% of the cases the patient and treating clinician score the patient status within 0.7 distance. CP_0.4 _Coverage Probability, proportion of cases within the Minimal Clinically Important difference of 0.4.

There were no differences in patient characteristics between patients with a score difference smaller than the MCID, and those larger than the MCID between patient and reviewing clinicians. No recurrent themes emerged from the interviews to explain low agreement.

## Discussion

This study uses a new method to assess the individual validity of a health status questionnaire. The method was applied in the management of COPD, with the Clinical COPD Questionnaire (CCQ) health status questionnaire. This study shows that there is a very good agreement in CCQ outcomes between the individual patient score and 20 reviewing clinicians who did not know the patient but scored the CCQ based on an in-depth interview. In combination with the previously known high reliability and stability, this confirms the validity of the CCQ at the individual patient level.

This new method to assess the validity of health status questionnaires in clinical practice is feasible for a short questionnaire, but requires much effort due to the patient interviews and the subsequent review by clinicians. However, by using transcripts of interviews with each individual patient, this method provides accurate and transparent information about the actual performance of a patient who is asked to complete a questionnaire in daily clinical practice. The use of qualitative methods to assess the individual accuracy of a questionnaire in routine practice provides more insight into individual validity than pure statistical methods assessing the internal consistency and stability of a questionnaire.

The validity of the CCQ at the group level has already been assessed [8,9,16,17]. In two of these studies internal stability and consistency was very high, thus meeting the requirements for individual use of the questionnaire [8,9]. However, in a recent study this high level was not met [16], possibly due to the different study population and methods used in that study. Nevertheless the high concordance between the results of the approach according to the standards as proposed by McHorney and Tarlov [3] and our new method using patient interviews, confirms the acceptability of this new method.

The high CCC indicates that there was no systematic error in measuring. The Blant and Altman plots also confirm this finding. The absence of a systematic error is in contrast to previous findings of a difference in patient-proxy ratings of quality of life [18] and differences in patient-clinician ratings [19]. Proxies and clinicians tend to rate the quality of life worse than the patients [18,20]. The domains of the questionnaires that cover emotions tend to differ more between proxies and patients than the domains measuring symptoms[18]. In the current studies we also see that the mental state domain shows the least concordance; however, there is no systematic under- or over-estimation compared to the patient's score.

For the Bland and Altman plots we chose a difference in scores of 0.4 (the MCID), as cut-off point for agreement. Over 60% of the 44 patient-reviewer scores differed less than the MCID. Of the patients-reviewer scores, 90% (the TDI_0.9_) were within 0.7 points. We chose the MCID because this difference in score would (according to the definition) potentially cause a clinician to change the management: "*the smallest difference in a score in the domain of interest which patients perceive as beneficial and which would mandate in the absence of troublesome side-effects and excessive costs a change in the patient's management*" [21]. Compared to others, we chose a very strict cut-off point. In Wilson's method for proxy ratings, a moderate difference is used [18]. For the CCQ, this moderate difference would be around 1.3 [13], which is far more than the 0.7 points in which 90% of scores were in the current study.

A limitation of this study is that this new method of assessing the validity of a health status questionnaire on the individual patient level could be improved by additional information about the individual patient's scoring stability and responsiveness to changes. The stability of scoring of the studied population could be assessed using the test-retest method. Although the test-retest reliability of the CCQ was very high in two studies [8,9], and so was the ability to measure treatment effects [17,22] we did not confirm this in the present group of patients.

The current study could not identify patient factors that were associated with low agreement between patient and reviewers. A possible explanation for low agreement in some individuals might be that most patients completed the questionnaire for the first time. During the interview, patients sometimes answered "*now I'm re-thinking about this, my score would have been*...". Although some patients with a low agreement gave the impression of lower intelligence, this could not be substantiated by a lower educational level.

In the present study we found a better agreement in scores between patients and the treating clinicians than others [19]. In contrast to other patient-proxy agreement studies, only two clinicians participated in the recruitment of the patients and the scoring of the CCQ, as the main research question was the patient-reviewer agreement. These two clinicians previously used the CCQ in their practice or in pulmonary rehabilitation programs. One clinician stated that he changed his history taking during the study, because he was unable to answer specific questions on multiple occasions, especially about the mental state domain. The experience in measuring health status and the change in history taking might contribute to the high agreement between the scores of the patient and treating clinician.

## Conclusion

In conclusion, this study shows that this new method to assess the individual validity of a questionnaire by using patient interviews is feasible, and confirms results from previous studies using statistical methods. Secondly, there seems to be a good validity of the CCQ on the individual patient level as established with this new methods. The CCQ can therefore be used in routine clinical practice to assess the health status of patients with COPD.

## Competing interests

The authors of this manuscript declare not to have any conflict of interest regarding this manuscript. None of the authors have any financial interests with any commercial entity that has interest in the subject- or outcome of this manuscript including consultancy, stock ownership, paid expert consultancy, or honoraria, patent application, as well as other forms of conflict of interest, including personal and academic issues. The authors to the best of their knowledge conducted the study and reported the conclusions independently without any interference from partial or full funding sources or other entities.

## Authors' contributions

JWHK: conception and design, acquisition of data, analysis and interpretation of data; initial drafting the manuscript and revising; gives final approval of the version to be published. HAMK: conception and design, interpretation of data; initial drafting the manuscript and revising; gives final approval of the version to be published. SLS: acquisition of data, analysis and interpretation of data; revising the manuscript; gives final approval of the version to be published. BdV: acquisition of data, analysis and interpretation of data; revising the manuscript; gives final approval of the version to be published. JJB: acquisition of data; revising the manuscript; gives final approval of the version to be published. PvH: acquisition of data; revising the manuscript; gives final approval of the version to be published. JHS: acquisition of data; revising the manuscript; gives final approval of the version to be published. HEPB: analysis and interpretation of data; revising the manuscript; gives final approval of the version to be published. TvdrM: conception and design, acquisition of data, analysis and interpretation of data; initial drafting the manuscript and revising; gives final approval of the version to be published.

## Authors' information

TvdrM had developed, with others, the CCQ
